# Interleukin (IL)‐1 in rat parturition: IL‐1 receptors 1 and 2 and accessory proteins abundance in pregnant rat uterus at term – regulation by progesterone

**DOI:** 10.14814/phy2.12866

**Published:** 2016-07-20

**Authors:** Tomohito Ishiguro, Jun Takeda, Xin Fang, Heather Bronson, David M. Olson

**Affiliations:** ^1^ Departments of Obstetrics and Gynecology, Physiology & PediatricsUniversity of AlbertaEdmontonCanada; ^2^ Departments of Obstetrics and GynecologyJuntendo University Faculty of MedicineTokyoJapan; ^3^ Departments of Obstetrics and GynecologyKoshigaya Municipal HospitalKoshigayaJapan

**Keywords:** IL‐1 receptor, IL‐1 receptor accessory protein, IL‐1 receptor accessory protein – brain dominant, parturition, uterus

## Abstract

The role of interleukin‐1 (IL‐1), a pro‐inflammatory cytokine, in parturition is typically noted by changes in its concentrations. Studying the expression of its receptor family, IL‐1 receptor (IL‐1R) 1, IL‐1R2, IL‐1R accessory protein (IL‐1RAcP), and its predominantly brain isoform, IL‐1RAcPb, during late gestation in the uterus in the Long‐Evans rat is another. We assessed changes in their mRNA and protein relative abundance in the uterus and compared IL‐1RAcP and IL‐1RAcPb mRNA abundance in uterus, cervix, ovaries, placenta, and whole blood of Long‐Evans rats during late gestation or in RU486 and progesterone‐treated dams using quantitative real‐time PCR and western immunoblotting. IL‐1R1, IL‐1RAcP, and IL‐1RAcPb mRNA abundance significantly increased in the uterus at delivery whereas IL‐1R2 mRNA abundance significantly decreased. IL‐1R1 protein increased at term and IL‐1R2 protein decreased at term compared to nonpregnant uteri. IL1‐RAcPb mRNA abundance was less than IL‐1RAcP, but in the lower uterine segment it was the highest of all tissues examined. RU486 stimulated preterm delivery and an increase in IL‐1R1 mRNA abundance whereas progesterone administration extended pregnancy and suppressed the increase in IL‐1R1. These data suggest that changes in uterine sensitivity to IL‐1 occur during late gestation and suggest another level of regulation for the control of delivery. The roles for IL‐1RAcP and IL‐1RAcPb need to be determined, but may relate to different intracellular signaling pathways.

## Introduction

It is widely accepted that the process of parturition utilizes inflammatory mediators (Christiaens et al. 2008; Fang et al. 2000; Liggins et al. 1977; Romero et al. 1989). Interleukin‐1 (IL‐1*α* and *β* forms) (Romero et al. 1991) and IL‐6, (Gomez‐Lopez et al. 2016; Robertson et al. 2010) for instance, are essential for on‐time delivery as IL‐6 gene deletion (Robertson et al. 2010) or receptor inhibition by the specific antibody, tocilizumab, (Wakabayashi et al. 2013) or administration of the naturally occurring IL‐1 receptor antagonist (IL‐1RA) (Romero and Tartakovsky 1992) or 101.10, a specific small peptide allosteric receptor antagonist to IL‐1R accessory protein (IL‐1R AcP) (Nadeau‐Vallée et al. 2015), delay term or preterm delivery in mouse models (Romero et al. 1989). Mouse models are exquisitely sensitive to lipopolysaccharide (LPS), a gram‐negative, toll‐like receptor (TLR) 4 agonist, lipoteichoic acid (LTA) a gram‐positive TLR2 agonist or IL‐1*β* induction of preterm delivery (Hirsch et al. 2002; Nadeau‐Vallée et al. 2015). In primate models, intra‐amniotic administration of IL‐1*β* or LPS leads to a robust inflammatory response, increases in myometrial contractions and cervical dilatation, and preterm delivery (Waldorf Adams et al. 2008, 2011; Sadowsky et al. 2000). Elevations in the concentrations of cytokines have been reported in both term and preterm labor with or without infection in gestational tissues, amniotic fluid, fetal membranes, myometrium, and cervix in several species (Bollapragada et al. 2009; Opsjøn et al. 1993; Osman et al. 2003; Romero et al. 1989, 1992). In rats, however, administration of IL‐1*β* or LPS has variable effects upon the timing of delivery (Bennett et al. 2000; Fang et al. 2000; Mitchell et al. 2005; Hirsch et al. 2009; Montalbano et al. 2013), and there are no reports of increased cytokines in rat uterine tissues or fluids at normal term delivery.

The IL‐1 family has 11 members of which IL‐1*α* and IL‐1*β* are the most studied in relation to parturition (Sims and Smith 2010). Interleukin‐1*β* expression is inducible and not constitutive, unlike IL‐1*α*. Interleukin‐1*β* is produced by hematopoietic cells such as dendritic cells, blood monocytes, T cells, and tissue macrophages (Kim et al. 2012; Pawelczyk et al. 2010; Schober et al. 2012), but can also be synthesized by uterine tissues and cells including myometrial smooth muscle (Montalbano et al. 2013; Osman et al. 2003; Xu et al. 2015; Young et al. 2002). The induction of its transcription is triggered by microbial stimuli, damage associated molecular patterns (DAMPs) from maternal tissues under progressively increasing physical stress as gestation advances, or lung surfactants released from the maturing fetus (Montalbano et al. 2013), via toll‐like receptor 4 (TLR4), or pro‐inflammatory cytokines, including itself (Pawelczyk et al. 2010). Receptors for IL‐1 are composed of extracellular immunoglobulin domains and the IL‐1 receptor domain in the cytoplasmic portion of cells (Sims and Smith 2010). Interleukin‐1*α*, IL‐1*β*, and IL‐1 receptor antagonist (IL‐1RA) have two receptors: IL‐1R1, which is biologically active, and IL‐1R2, which lacks a signalling‐competent cytoplasmic tail and acts as a decoy. Interleukin‐1R1 is activated once the IL‐1RAcP binds IL‐1 and IL‐1R1 to form a complex that activates intracellular adapter molecules (Arend et al. 2008). Interleukin‐1RAcP has an alternatively spliced isoform that varies in the C‐terminal region termed IL‐1RAcPb because it is primarily expressed in the central nervous system (CNS) (Huang et al. 2011; Smith et al. 2009).

Given the apparently divergent responses between mice and rats to IL‐1*β* and its complex relationship with its receptors and accessory proteins, we hypothesized that another level of regulation of IL‐1*β* action at the receptor level exists in relation to its role in parturition, that is, regulation by progesterone. We therefore explored the relative abundance of IL‐1 receptors and accessory proteins in Long‐Evans rats and reproductive tissues in late gestation and compared the roles of IL‐1*β*, progesterone and its receptor antagonist, RU486, on their expression as well as the timing of delivery.

## Materials and Methods

### Animals

The University of Alberta Health Sciences Animal Policy and Welfare Committee approved all animal experiments. We used Long‐Evans rats for consistency with our other studies including the effects of stress upon gestation length (Yao et al. 2014). Female virgin rats (Charles River, Portage, MI) were housed with male Long‐Evans rats for 3 h (from 14:00 to 17:00) to impregnate rats. The pregnant rats weighing 250–350 g were transferred from Charles River to our facility at gestational day (GD) 14. They were individually housed under standard environmental conditions: in a 12 h light: 12 h dark cycle in the University of Alberta Health Sciences Laboratory Animal Services and were fed food and water ad libitum. Start of GD 1 was defined at 15:30, corresponding to the midpoint of the mating time. Under these conditions, the average time for labor to start in normal pregnant rats (nontreated) was early GD 22 (mean time 528.1 h). In this study, parturition was defined as rats delivering at least one pup.

### Experimental design

#### IL‐1β‐treated group

To evaluate whether the administration of IL‐1*β* might induce preterm labor in rats, pregnant rats were treated with increasing amounts of IL‐1*β* (5, 10, 20, and 100 *μ*g in 0.3 mL sterile saline, i.p.; Prospec‐Tany TechnoGene Ltd., Ness Ziona, Israel) or vehicle on GD 17. The rats in each group were killed on GD 20, 21 and during labor, and the tissue samples (upper uterus, lower uterus, and plasma) were collected for mRNA analysis or protein analysis. In all treatments, agents were administered at 10:00.

#### RU486‐treated group

The pregnant rats were randomly treated with either the progesterone receptor antagonist, RU486 (mifepristone, 11*β*‐[4‐(dimethylamino)phenyl]‐17*β*‐hydroxy‐17‐(1‐propynyl)‐estra‐4,9‐dien‐3‐one; Cayman Chemical Company, Ann Arbor, MI) at 10 mg/kg in 0.4 mL corn oil (s.c.) containing 10% ethanol or vehicle on GD 17 (Mitchell and Lye 2002; Shynlova et al. 2004, 2005, 2007a,b, 2008). The rats in both groups (each *n* = 4–5) were killed at 12 h and 24 h after drug or vehicle administration, and the uterine tissue samples were collected for mRNA analysis.

#### Progesterone‐treated group

Pregnant rats were treated daily beginning on GD19 with P4 (medroxyprogesterone acetate, 16 mg/kg/day, subcutaneous injection in 0.4 mL corn oil containing 10% ethanol (s.c.); Sigma‐Aldrich, Oakville, ON) (Lan et al. 1999; Romero et al. 2006; Sadowsky et al. 2000; Schober et al. 2012; Shynlova et al. 2004, 2005). The P4‐treated dams were killed on GD 21 and 23 (*n* = 4–5 at each time point), and the tissue samples (upper uterus and lower uterus) were collected.

### Tissue and plasma collection

The animals were killed according to the approved local policy with a lethal dose of isoflurane (Halocarbon Products Corporation, Atlanta, GA) by inhalation in a large beaker. Uterine horns were divided into three equal‐length segments: upper, middle, and lower, and the upper and lower segments were examined. All tissue samples (upper uterus, lower uterus, cervix, ovaries, placenta, and blood) were collected at 9:00 on GD 17, 20, and 21 or during labor immediately following the delivery of the first pup (*n* = 3–7 animals/group). Tissue samples were washed in phosphate buffered saline buffer (137 mmol/L NaCl, 2.7 mmol/L KCl, 10 mmol/L Na_2_HPO_4_, 1.8 mmol/L KH_2_PO_4_), flash‐frozen in liquid nitrogen and stored at −80°C. For each gestational day, tissue samples were collected, blotted, immediately frozen in liquid nitrogen and stored at −80°C.

### Quantitative real‐time PCR

Total RNA was isolated from the frozen tissue samples using Qiagen RNeasy kits (Qiagen, Valencia, CA) with TRIzol Reagent (Life Technologies, Carlsbad, CA). Complementary DNA (cDNA) was synthesized from 500 ng total RNA by reverse transcription using the qScript^™^ cDNA SuperMix (Quanta BioSciences, Gaithersburg, MD). The resultant cDNA was used as a template to perform real‐time polymerase chain reaction (RT‐PCR) using gene‐specific primers summarized in Table 1. Quantitative RT‐PCR was conducted using the iCycler (Bio‐Rad, Hercules, CA) with the SYBR Green FastMix (Quanta BioScience) and specific sets of primers (Table 1) (Parker et al. 2000; Taishi et al. 2012) as previously described (Gomez‐Lopez et al. 2013). Reaction mixtures contained 1 *μ*L cDNA (50 ng/*μ*L), 10 *μ*L** **SYBR Green FastMix, 0.5 *μ*L forward primer (10 *μ*mol/L), 0.5 *μ*L reverse primer (10 *μ*mol/L), and sterile water in a total reaction volume of 20 *μ*L. Reactions were incubated at 95°C for 10 min and then for 40 cycles of 95°C for 15 sec, and each sample was annealed (Table 1) for 1 min. Melt curve analysis was performed following amplification to verify that amplification of nonspecific products was not present. There was no amplification of nonspecific products with each set of primers. All protocols were performed according to the manufacturer's instructions. The expression of target genes was normalized to that of *Cyclophilin a* as the housekeeping gene. The abundance of *Cyclophilin a* remained constant in tissue through the period tested.

**Table 1:** Primers used in real‐time polymerase chain reaction (RT‐PCR)

Gene	Primer sequences	Annealing temperature (°C)	NCBI reference sequences^a^
*Il1a*			
Forward	5′‐ GGC TAA GTT TCA ATC AGC CCT TT ‐3′	60	NM_017019.1
Reverse	5′‐ AGG TGC TGA TCT GGG TTG GAT ‐3′		
*Il1b*			
Forward	5′‐ CTC AAT GGA CAG AAC ATA AGC C ‐3′	51	NM_031512.2
Reverse	5′‐ GGT GTG CCG TCT TTC ATC A ‐3′		
*Ptgs2*			
Forward	5′‐ CCT TGA ACA CGG ACT TGC TCA C ‐3′	60	S67722.1
Reverse	5′‐ TCT CTC TGC TCT GGT CAA TGG A ‐3′		
*Ptgfr*			
Forward	5′‐ CTG GCC ATA ATG TGC GTC TC ‐3′	60	NM_013115.1
Reverse	5′‐ TGT CGT TTC ACA GGT CAC TGG ‐3′		
*Il1r1*			
Forward	5′‐ CCT GTG ATT ATG AGC CCA CG ‐3′	58	NM_013123.3
Reverse	5′‐ CGT GTG CAG TCT CCA GAA TAT G ‐3′		
*Il1r2*			
Forward	5′‐ AGA TGA GCC AAG GAT GTG GG ‐3′	62	NM_053953.1
Reverse	5′‐ ATC AAT AGG CGT GTG GGG TCT ATA CCA CTG TAT CTT TCC A ‐3′		
*Il1rap*			
Forward	5′‐ GGG CAA CAT CAA CGT CAT TTT AG ‐3′	68	NM_012968.1
Reverse	5′‐ CAG CTC TTT CAC CTT CAA GTC CTT ‐3′		
*Il1rapb*			
Forward	5′‐ GGA GTT TAA GCT GGG TGT CAT GT ‐3′	68	NM_001167840.1
Reverse	5′‐ TGC TCA AGC GGA CGG TAC T ‐3′		
*Cyclophilin a*			
Forward	5′‐ CAC CGT GTT CTT CGA CAT CAC ‐3′	60	NM_017101.1
Reverse	5′‐ CCA GTG CTC AGA GCA CGA AAG ‐3′		

a NCBI, National Center for Biotechnology Information (http://www.ncbi.nlm.nih.gov).

### Enzyme‐linked immunosorbent assay

The plasma concentrations of progesterone were measured directly by enzyme‐linked immunosorbent assay (ELISA) using a Progesterone Enzyme Immunoassay Kit (EIA‐1561; Both intra‐ and inter‐assay coefficient <10%; DRG International Inc., Springfiled, NJ), according to manufacture's instruction. To adjust the concentrations of every sample to the range of the assay (between 0 and 40 ng/mL), each sample was diluted 25‐fold with the buffer provided in the kit.

### Statistical analyses

All data were tested (Bartlett's Test) for homogeneity of variance and were found to be normally distributed. Data are presented in box and whisker plots as the median −/+ 25% and 75% quartiles and range. Comparisons were made using the Student's *t*‐test or one‐way analysis of variance (ANOVA) with Dunn's multiple comparison test or Tukey's multiple comparison test or two‐way ANOVA with Sidak's multiple comparison test. All statistics were performed with GraphPad Prism Version 6.0a software (GraphPad Software, San Diego, CA); *P *< 0.05 was considered statistically significant.

## Results

### The effect of IL‐1β administration on timing of delivery, uterine mRNA and protein expression of Il1a, Il1b, Ptgs2, and Ptgfr

**Figure 1:**
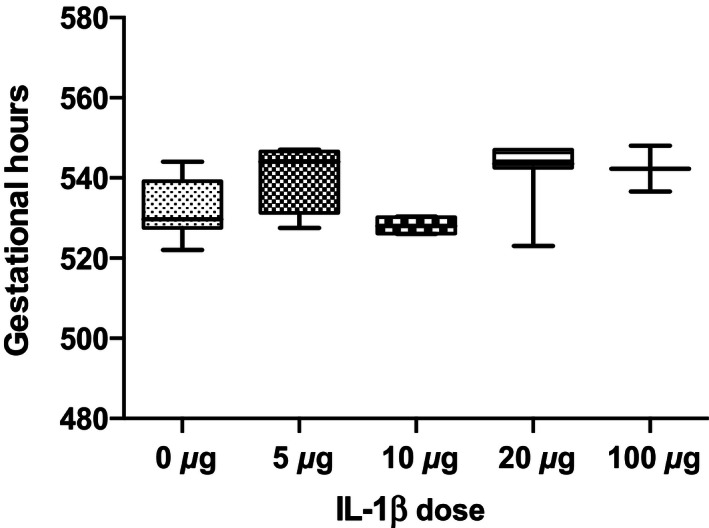
Effect of increasing amounts (5–100 *μ*g) of IL‐1*β* or vehicle administration on gestational length (*n* = 2–7 dams at each concentration). There is no effect of IL‐1*β* on the length of gestation.

**Figure 2:**
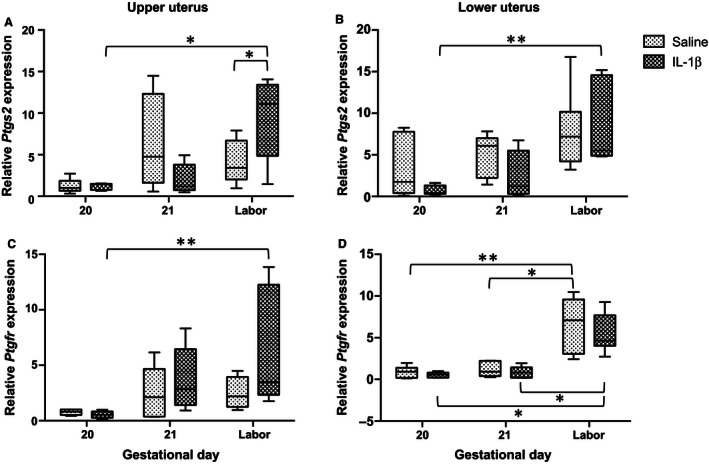
Gestational age‐dependent changes in *Ptgs2* (A, B) and *Ptgfr* (C, D) mRNA abundance in late gestation in rat uterus and effect of IL‐1*β* administration (20 *μ*g, i.p.) or vehicle in upper (A, C) and lower (B, D) uterine segments (*n* = 3–7 dams). There were gestational age‐dependent increases in mRNA abundance of *Ptgs2* in upper (*P < 0.05) and lower uterus (**P < 0.01) and of *Ptgfr* in upper (**P <* *0.01) and lower uterus (**P < 0.01). IL‐1*β* administration stimulated a late increase in laboring dams in *Ptgs2* mRNA abundance in upper uterus (*P < 0.05).

**Figure 3:**
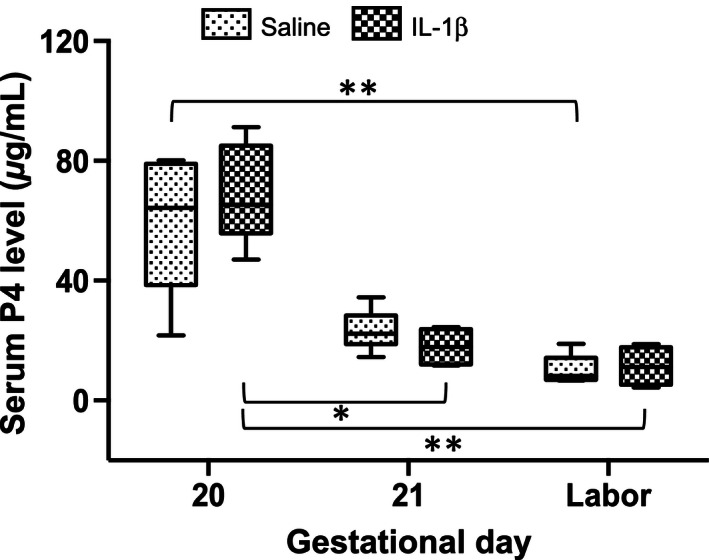
Circulating plasma progesterone (P4) concentrations in late gestation and effect of IL‐1*β* administration (20 *μ*g, i.p.) or vehicle in rats (*n* = 4–7). Progesterone decreased in late gestation (**P < 0.01) and IL‐1*β* had no effect on these concentrations.

In order to determine whether the Long‐Evans pregnant dam responds to IL‐1*β* with decreased length of gestation and increased uterine cytokine expression similar to mice (Nadeau‐Vallée et al. 2015), we administered IL‐1*β* (5–100 *μ*g/dam, intraperitoneally) on GD17 (Paris et al. 2011). We were unable to stimulate preterm delivery in rats (Fig. 1). While we observed gestational age‐dependent increases in abundance of *Ptgs2* mRNA in upper (*P < 0.05) and lower uterus (**P < 0.01) (Fig. 2A and B) and of *Ptgfr* in upper (**P < 0.01) and lower uterus (*P < 0.05, **P <* *0.01) (Fig. 2C and D), neither was sensitive to IL‐1*β* administration (20 *μ*g, i.p.) prior to delivery. Only at delivery did IL‐1*β* administration lead to an increase in *Ptgs2* in upper uterus (Fig. 2A, *P < 0.05). The late gestation fall in plasma progesterone (Fig. 3; *P <* *0.05, **P <* *0.01) was not affected by IL‐1*β* administration (Fig. 3).

As the rat was relatively unresponsive to IL‐1*β* in ways the mouse was responsive, we studied the expression of IL‐1 receptors during late gestation.

### The abundance of Il1r1, IL1r2, Il1rap, and Il1rapb in the uteri of parturient rats

**Figure 4:**
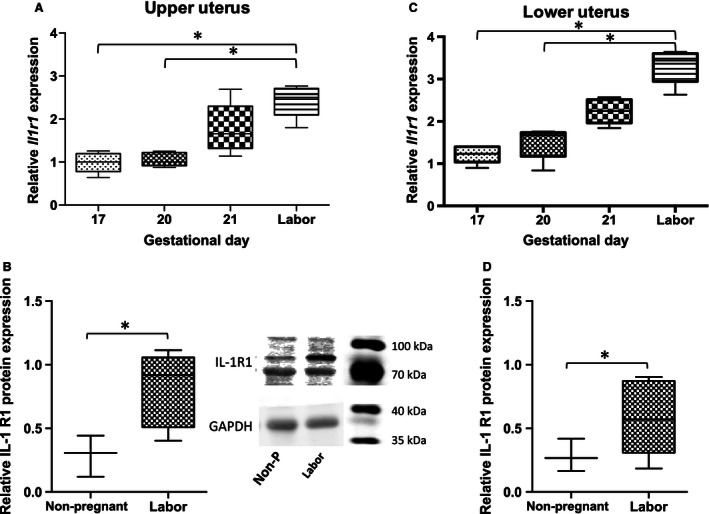
Relative abundance of IL‐1R1 in late gestation rat uterus. A, C *Il1r1* mRNA abundance increases at labor versus GD17 and GD20 in upper uterus and lower uterus (*P <* *0.05). B, D IL1‐R1 protein mass increases at labor compared to nonpregnant in upper uterus and lower uterus (*P *<* *0.05); (mRNA,* n* = 5 dams at each time point; protein, *n* = 3–6 dams). GD, gestational day.

Figure 4A and B demonstrate that *Il1r1* relative mRNA abundance in both upper and lower uterine segments increased from GD17 and GD20 to Labor (upper uterus; *P < 0.05, lower uterus; *P < 0.05). These increases in mRNA abundance were accompanied by significant increases in protein relative abundance in upper and lower uteri from term animals compared to nonpregnant uteri (each *P < 0.05) (Fig. 4B and D).

**Figure 5:**
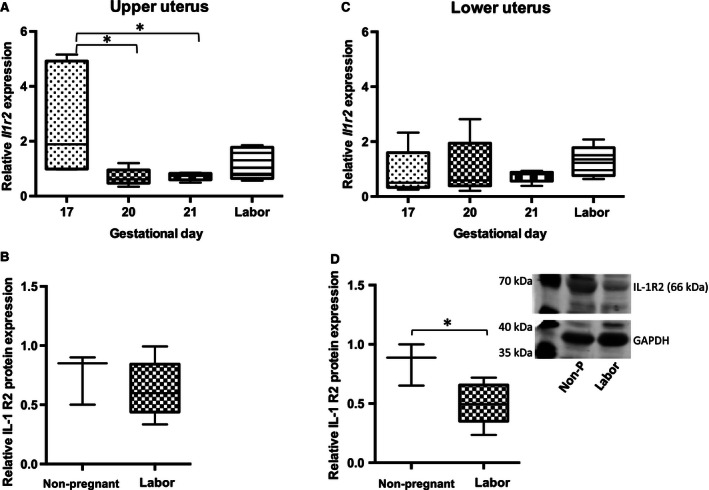
Relative abundance of IL‐1R2 in late gestation rat uterus. A, C *Il1r2* mRNA abundance decreases on GD20 and GD21 versus GD 17 in upper uterus (*P <* *0.05) but there is no change in lower uterus. B, D median IL‐1R2 protein abundance decreases in laboring versus nonpregnant upper uterus (NS) and lower uterus (*P <* *0.05); (mRNA,* n* = 5 dams at each time point; protein, *n* = 3–6 dams).GD, gestational day.

In contrast to *Il1r1* mRNA expression, the mRNA expression of *Il1r2* in the upper uterus significantly decreased before parturition (Fig. 5A and C; GD17 vs. GD20 and 21** ^*^ *P* < 0.05). The protein abundance of IL‐1R2 decreased in both upper and lower uterus between nonpregnant and laboring dams, but was significantly different only in lower uterus (Fig. 5B and D; *P <* *0.05).

**Figure 6:**
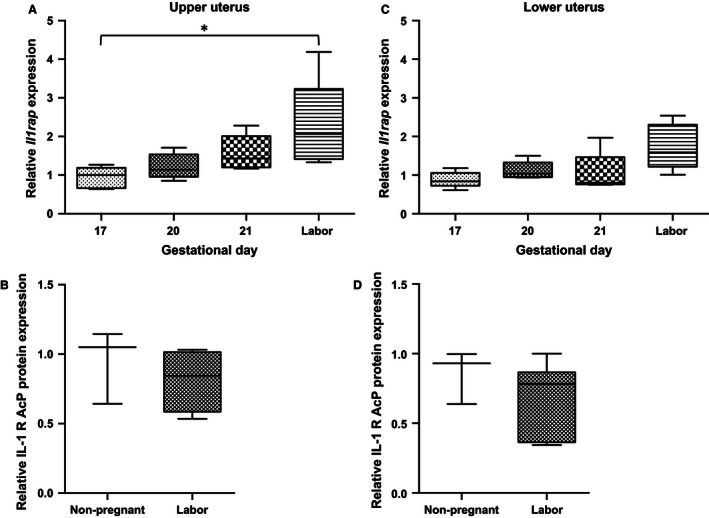
Relative abundance of IL‐1RAcP in late gestation rat uterus. (A, C) *Il1rap* mRNA abundance increases at labor versus GD17 in upper uterus (*P <* *0.05) and there is nonsignificant trend to increase lower uterus. (B, D) median IL‐1RAcP protein abundance is not different in laboring versus nonpregnant dams; (mRNA,* n* = 5 dams at each time point; protein, *n* = 3–6 dams).

The mRNA relative abundance of *Il1rap* gradually increased from GD17 to parturition in both upper and lower uterine segments, but the increase was significant only in the upper uterus (Fig. 6A and C; *P < 0.05). There were no changes in the protein abundance for IL1rap between nonpregnant and laboring uteri (Fig. 6B and D).

**Figure 7:**
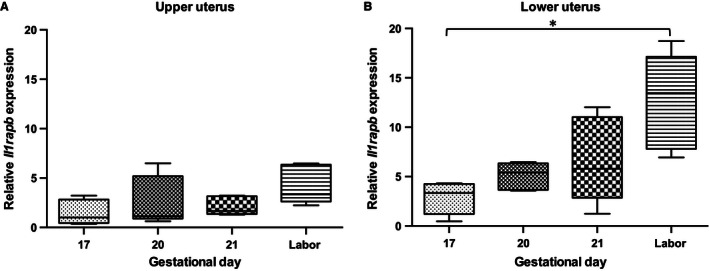
Relative abundance of IL‐1RAcPb in late gestation rat uterus. (A, B) median *Il1rapb* mRNA abundance increases in upper (NS) and lower uterus at labor versus GD17 (*P <* *0.05); (*n* = 5 dams at each timepoint). GD, gestational day.

Figure 7 describes the fourfold increase in the mRNA abundance of *Il1rapb* in the lower uterine segment from GD17 to parturition (Fig. 7B; *P < 0.05). Based upon PCR cycles, it is estimated that the relative abundance of *Il1rap* mRNA is about 10‐fold more than that of *Il1rapb*. There was no difference in uterine protein mass in IL‐1RAcP between nonpregnant and delivering dams (data not shown; there are no commercially available antibodies to IL‐1RAcPb).

### Effects of RU486 and progesterone on IL‐1RI mRNA relative abundance in the uterus of pregnant rats

**Figure 8:**
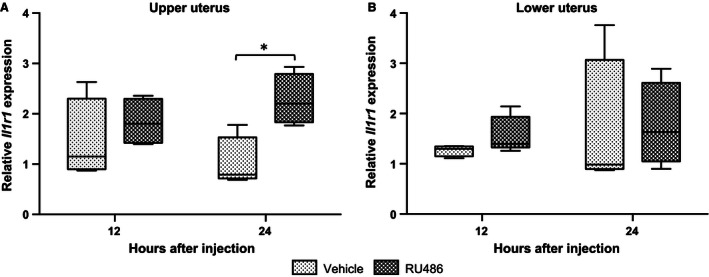
The effect of RU486 (A upper uterus, B lower uterus) on *Il1r1* relative mRNA abundance. Dams were treated with vehicle (*n* = 5 at each time point) or RU486 (*n* = 4 at each time point). RU486 increases *Il1ra* mRNA abundance in upper uterus 24 h after administration (*P <* *0.05). GD, gestational day.

Figure 8 describes the effects of RU486 administration (10 mg/kg, s.c.) on the mRNA abundance of *Il1r1* in the uterus 12 h and 24 h after injection on GD17. Delivery occurred on GD18, 24.04 ± 0.69 h (*n* = 6) after RU486 administration. None of the vehicle‐administered dams were in labor. Overall there were trends toward increasing abundance of Il1r1 abundance at 12 and 24 h in upper and lower uteri, but the only significant increase of *Il1r1* mRNA abundance was in the upper uterine segment at 24 h after RU486 administration (Fig. 8A; *P < 0.05). There was no difference in the mRNA abundance of *Il1r2*,* Il1rap,* or *Il1rapb* due to RU486 treatment (data not shown).

**Figure 9:**
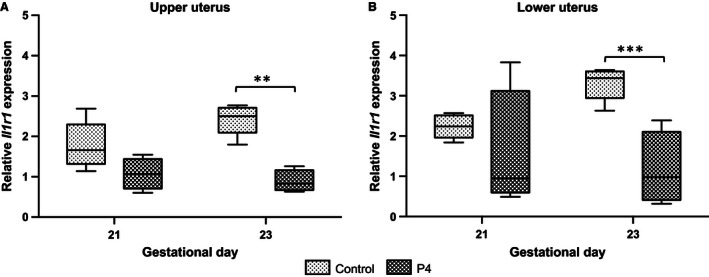
The effect of progesterone (P4) (A upper uterus, B lower uterus) on *Il1r1* relative mRNA abundance in the myometrium. Dams were treated with vehicle (*n* = 5 at each time point) or P4 (*n* = 4 at each time point). P4 significantly decreased *Il1r1* mRNA abundance in upper and lower uterus on GD23 (**P <* *0.01, ***P <* *0.001). GD, gestational day.

We observed opposite effects with progesterone administration (16 mg/kg/day). Delivery had not occurred by GD23 (1 day late) and progesterone decreased *Il1r1* mRNA abundance in both upper and lower uterine segments on GD23 (Fig. 9A and B; **P <* *0.01 and ***P <* *0.001) compared to vehicle‐treated controls dams. Progesterone treatment had no significant effect upon *Il1r2*,* Il1rap*, and *Il1rapb* mRNA abundance (data not shown).

### Il1rap and Il1rapb relative abundance in reproductive and blood tissues of rats during late gestation

**Figure 10:**
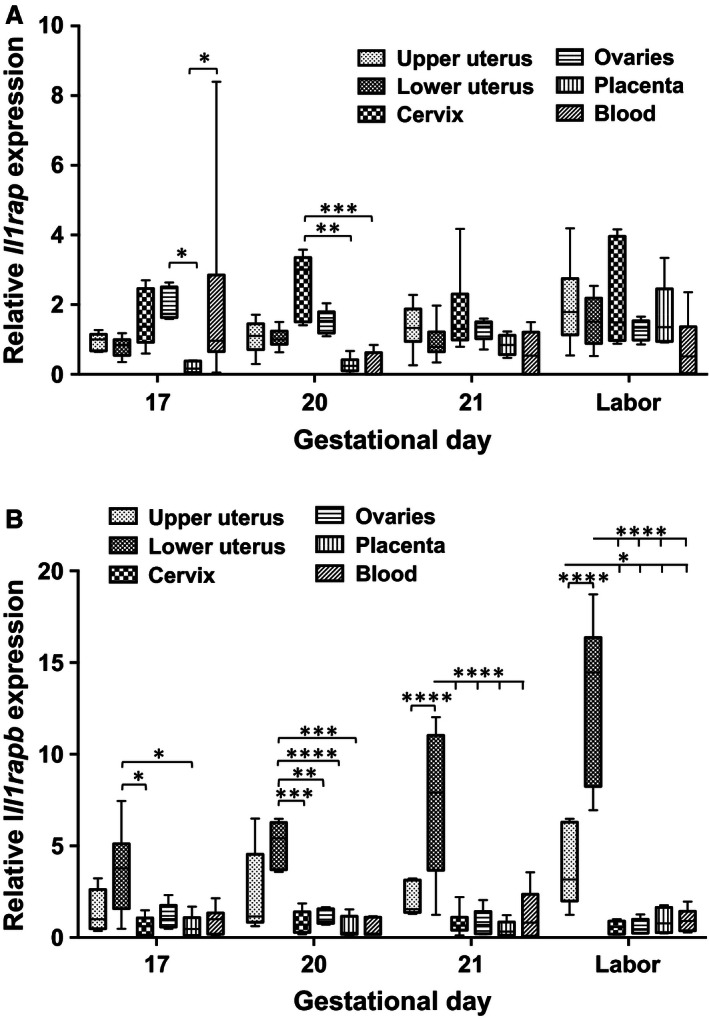
(A) The relative abundance of *Il1rap* and *Il1rapb* in reproductive tissues and blood. The relative mRNA abundance of *Il1rap* in upper and lower uterine segments, cervix, ovaries, placenta, and whole blood of rats during late gestation (*n* = 5 at each time point, *P <* *0.05, **P <* *0.01, ***P <* *0.001). (B) The relative mRNA abundance of *Il1rapb* in upper and lower uterine segments, cervix, ovaries, placenta, and whole blood of rats during late gestation. There was a significant increase in lower uterus with approaching delivery (*n* = 5 at each time point, *P <* *0.05, **P <* *0.01, ***P <* *0.001).

All tissue samples (upper and lower uterus, cervix, ovaries, placenta and blood [leukocytes]) expressed *Il1rap* mRNA (Fig. 10A). Abundance levels did not change with increasing gestational age; there were tissue level differences at GD17 and GD18 (*P <* *0.05, **P <* *0.01, ***P <* *0.001). In contrast, there were several remarkable differences regarding the relative abundance of *Il1rapb* mRNA in the lower uterine segment in contrast to the other tissues: it was considerably higher than in the other tissues examined, and it increased significantly in late gestation (Fig. 10B; *P <* *0.05, **P <* *0.01, ***P <* *0.001, ***^*^ *P *<* *0.0001).

## Discussion

The inability of IL‐1*β* to stimulate preterm delivery or expression of *Il1a*,* Il1b,* or *Ptgfr* plus inconsistent stimulation of *Ptgs2* mRNA expression until late in gestation suggests a relative insensitivity to IL‐1*β* until very late in gestation. Hence, our examination of the timing of expression for IL‐1 receptors in late gestation is justified and reveals both an involvement of IL‐1*β* in parturition and a potential regulatory role for IL‐1 receptors in dictating the timing of labor in pregnant Long‐Evans rats.

Our results suggest that the reciprocal nature of changes in IL‐1R1 and IL‐1R2 in the uterus toward parturition is related to the initiation of labor. Presumably the uterine myometrium becomes more sensitive to IL‐1*β* and there is less decoy receptor present to bind and thereby neutralize IL‐1*β*. One regulator of this change is progesterone, which decreases in late gestation (Fig. 3). This can explain our observation of the insensitivity of the rat to administration of IL‐1*β* prior to the very end of gestation. The effects of RU486 or P4 on rat uterine IL‐1R1 are similar to their effects on the expression of monocyte chemoattractant protein‐1 (MCP‐1/CCL‐2) (Shynlova et al. 2005), oxytocin receptor, and prostaglandin F2*α* (PGF2*α*) (Fang et al. 1997). This result is compatible with the hypothesis that progesterone inhibits the inflammatory response to maintain uterine quiescence (Lan et al. 1999).

Other regulators of *Il1r1* are IL‐1*β* and PGF2*α*. IL‐1*α* and IL‐1*β* induce the expression of oxytocin receptors (Terzidou et al. 2006), *Ptgs2* (Bartlett et al. 1999), and NF*κ*B in human myometrium (Terzidou et al. 2006), which have a crucial role in parturition. Recently, we demonstrated that IL‐1*β* and PGF2 induce *Il1r1* in cultured human myometrial smooth muscle cells (Verstraeten et al. 2015), and IL‐1*β* induces the expression of *Il1r1* in mouse uterus (Nadeau‐Vallée et al. 2015). IL‐1*β* administration predictably induces preterm birth in mice (Romero et al. 1991) and nonhuman primates (Sadowsky et al. 2000).

IL‐1*β* (and IL‐1*α*) bind to IL‐1R1 which then complexes with IL‐1RAcP (Sims and Smith 2010). The third ligand that is bound by IL‐1R1 is IL‐1RA. When IL‐1RA is administered to mice, it prevents the induction of preterm delivery by IL‐1*β* (Romero and Tartakovsky 1992), suggesting that IL‐1RA competes with IL‐1*β* for binding to IL‐1RI. In addition to IL‐1RA, the other naturally occurring inhibitor of IL‐1*β* and IL‐1R1 binding is IL‐1R2, a decoy receptor. It can exist in a membrane‐bound form or a soluble form that binds both IL‐1*β* and IL‐1RAcP but does not signal. Our observation that *Il1r2* decreases relative abundance near term while the expression of *Il1r1* and *Il1rap* increases suggesting an intriguing mechanism for effecting parturition. We do not know what the signal for decreased *Il1r2* is in late gestation; it apparently is not progesterone according to our data.

We reported that uterine mRNA abundance of IL‐1RAcPb increases near parturition particularly in the lower segment. Previously, Smith et al. (2009) reported that *Il1rapb* was nearly exclusively expressed in the CNS of humans. Not only did we find it in the uterus, we also found its expression in the cervix, ovaries, placenta, and blood but in much lower abundance levels than that in the uterus. The role of IL‐1RAcPb in parturition is unknown. They suggested that IL‐1RAcPb modulates the responses to IL‐1 in the CNS (Smith et al. 2009). In other work, this group indicates that IL‐1*β* complexed with IL‐1R1 and IL‐1RAcPb does not activate NF*κ*B but rather affects SRC phosphorylation and MAPK activity (Huang et al. 2011). This is consistent with the work of MacIntyre et al. (2014)who reported that NF*κ*B activation is not required for inflammation (LPS)‐driven preterm birth but that AP‐1/JNK activation is sufficient for delivery. Perhaps IL‐1RAcPb may be the more important accessory protein in the uterus for IL‐1*β*‐driven parturition events. Alternatively, it may serve as a cell adhesion molecule as suggested by Yoshida et al. (2012) in the brain. Further investigation will be required to define its role.

These data suggest that additional levels of regulation involving interleukin receptors and accessory proteins are required for the initiation of parturition, especially in the rat. One potential regulator of *Il1r1, Il1r2,* and *Il1racb* other than progesterone might be stress (Paris et al. 2011; Yao et al. 2014). With an improved understanding of physiological and cellular mechanisms leading to birth, more opportunities for identifying biomarkers that predict women at risk of preterm birth and more targets for therapeutic intervention to delay preterm delivery and to prolong and improve pregnancy outcomes may present themselves. In the long‐term, these data may lead to better diagnostics and therapeutics.

## Conflicts of Interest

The authors declare no conflicts of interest.
